# [μ-2,2′-Dimethyl-2,2′-(*p*-phenyl­ene)diprop­yl]bis­[chloridobis(2-methyl-2-phenyl­prop­yl)tin(IV)]

**DOI:** 10.1107/S1600536810011670

**Published:** 2010-04-10

**Authors:** Chunliu Sun, Chong Liang, Wanli Kang, Dongsheng Zhu

**Affiliations:** a Institute of Porous Flow and Fluid Mechanics, CNPC & Chinese Academy of Sciences, Langfang 065007, People’s Republic of China; b Research Institute of Petroleum Exploration and Development–Langfang, Langfang 065007, People’s Republic of China; c College of Petroleum Engineering, China University of Petroleum, Qingdao 266555, People’s Republic of China; d Faculty of Chemistry, Northeast Normal University, Changchun 130024, People’s Republic of China

## Abstract

The mol­ecular structure of the title compound, [Sn_2_(C_10_H_13_)_4_(C_14_H_20_)Cl_2_], is a binuclear centrosymmetric complex, in which the Sn atoms are four-coordinated by three C atoms and one Cl atom in a distorted tetra­hedral geometry.

## Related literature

For general background to organotin compounds, see: Chandrasekhar *et al.* (2002 ▶); Wu *et al.* (2009 ▶); For related structures, see: Tarassoli *et al.* (2002 ▶).
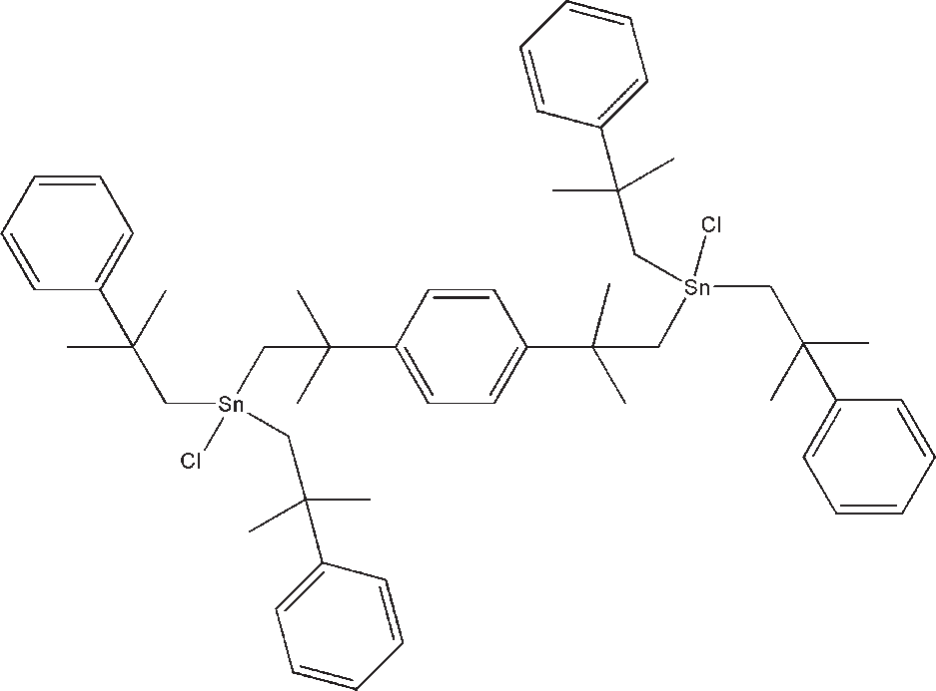


## Experimental

### 

#### Crystal data

[Sn_2_(C_10_H_13_)_4_(C_14_H_20_)Cl_2_]
*M* *_r_* = 1029.40
Monoclinic, 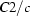

*a* = 15.0769 (19) Å
*b* = 17.773 (2) Å
*c* = 18.914 (2) Å
β = 94.674 (2)°
*V* = 5051.4 (11) Å^3^
*Z* = 4
Mo *K*α radiation
μ = 1.13 mm^−1^
*T* = 185 K
0.34 × 0.32 × 0.29 mm

#### Data collection

Bruker SMART APEX CCD area-detector diffractometer
Absorption correction: multi-scan (*SADABS*; Sheldrick, 1996 ▶) *T*_min_ = 0.688, *T*_max_ = 0.721
14065 measured reflections
4976 independent reflections
3284 reflections with *I* > 2σ(*I*)
*R*_int_ = 0.073

#### Refinement

*R*[*F* ^2^ > 2σ(*F* ^2^)] = 0.056
*wR*(*F* ^2^) = 0.113
*S* = 1.02
4976 reflections
262 parameters
H-atom parameters constrained
Δρ_max_ = 0.78 e Å^−3^
Δρ_min_ = −0.42 e Å^−3^

### 

Data collection: *SMART* (Bruker, 1998 ▶); cell refinement: *SAINT* (Bruker, 1998 ▶); data reduction: *SAINT*; program(s) used to solve structure: *SHELXS97* (Sheldrick, 2008 ▶); program(s) used to refine structure: *SHELXL97* (Sheldrick, 2008 ▶); molecular graphics: *SHELXTL-Plus* (Sheldrick, 2008 ▶); software used to prepare material for publication: *SHELXTL-Plus*.

## Supplementary Material

Crystal structure: contains datablocks global, I. DOI: 10.1107/S1600536810011670/pb2026sup1.cif

Structure factors: contains datablocks I. DOI: 10.1107/S1600536810011670/pb2026Isup2.hkl

Additional supplementary materials:  crystallographic information; 3D view; checkCIF report

## Figures and Tables

**Fig. 1.**
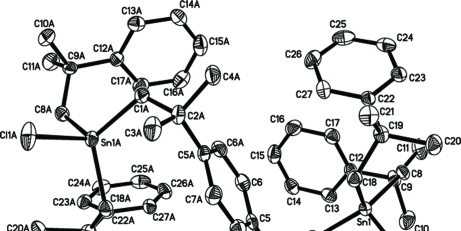
The molecular structure of (I), showing 30% probability displacement ellipsoids. H atoms have been omitted for clarity. Scheme 1. The chemical structure diagram of (I). Scheme 2. The reaction scheme for synthesis of (I).

**Fig. 2.**
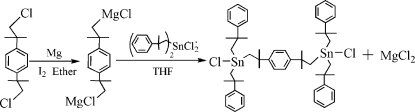
The formation of the title compound.

## supplementary crystallographic information

### Comment

The increasing interest in organotin compounds that has arisen in the last few decades is attributed to their significantly important biological properties like antiviral and anticancer agents, in vitro antibacterial and antifungal agents, wood preservatives and pesticides, etc. (Chandrasekhar *et al.*, 2002; & Wu *et al.*, 2009). Therefore, synthesis of new organotin compounds with different structural features will be beneficial in the development of pharmaceutical organotin and in other properties and application. herein, we present the synthesis and crystal structure of the title compound (I).

The structure of the title compound (Fig.1) consists of two symmetry equivalent tin moieties, where the tin atoms are tetrahedrally coordinated by the three C atoms and one Cl atom. The bond lengths for Sn(1)—C(1), Sn(1)—C(8) and Sn(1)—C(18) are 2.146 (5), 2.152 (5) and 2.149 (5) Å, respectively, which are slightly shorter than the Bz_3_Sn(EtACDA) reported by Tarassoli *et al.* (Tarassoli *et al.*, 2002). Around the tin, the angles C(1)—Sn(1)—C(8), C(1)—Sn(1)—C(18) and C(8)—Sn(1)—C(18) are wider while the C(1)—Sn(1)—Cl(1), C(8)—Sn(1)—Cl(1) and C(18)—Sn(1)—Cl(1) are narrower than the ideal tetrahedral angle. Thus, the environment of tin is best described as distorted tetrahedral.

### Experimental

A small iodine grain, magnesium powder(0.24 g 10 mmol), and 1,4-bis(1- chloro-2-methylpropan-2-yl)benzene (0.52 g, 2 mmol) were added to 2 ml of anhydrous ether under stirring. The reaction mixture is then heated to 50 –60°C by hot-water bath and maintained slight boiling state. When the purplish red of iodine disappeared, which indicated the reaction were initiated, the hot-water bath was removed. The reaction were keeping the slight boiling state, then a solution of 1,4-bis(1-chloro-2-methylpropan- 2-yl)benzene (2.59 g, 10 mmol) in 10 ml anhydrous ether were added dropwise. After finished, the mixture was refluxed for 1 h to allow magnesium to proceed to completion, then cooled to 0–5°C by ice-salt bath. A solution of dichlorobis(2-methyl-2-phenylpropyl)stannane (4.56 g,10 mmol) in 15 mL THF were then added dropwise. After finished, the ice-salt bath was removed, and the reaction mixture were stirred for 0.5 h at room temprature then refluxed for another 1.5 h. Finally, the mixture were again cooled to 0°C, and acidified by dropwise adding a solution containing 2.5 g fuming HCl and 15 ml water. The layers were separated, the organic phase was dried over anhydrous calcium chloride. Following filtration and evaporation of the solvent, the residue was recrystallized by THF and the colorless block crystals of (I) were abtained.

### Refinement

All H atoms on C atoms were positioned geometrically (C—H = 0.93 Å) and refined as riding, with U_iso_(H)=1.2U_eq_(carrier).

### Crystal data

**Table:**

[Sn_2_(C_10_H_13_)_4_(C_14_H_20_)Cl_2_]	*F*(000) = 2120
*M**_r_* = 1029.40	*D*_x_ = 1.354 Mg m^−^^3^
Monoclinic, *C*2/*c*	Melting point: not measured K
Hall symbol: -C 2yc	Mo *K*α radiation, λ = 0.71073 Å
*a* = 15.0769 (19) Å	Cell parameters from 4976 reflections
*b* = 17.773 (2) Å	θ = 1.8–26.1°
*c* = 18.914 (2) Å	µ = 1.13 mm^−^^1^
β = 94.674 (2)°	*T* = 185 K
*V* = 5051.4 (11) Å^3^	Block, colorless
*Z* = 4	0.34 × 0.32 × 0.29 mm

### Data collection

**Table:**

Bruker SMART APEX CCD area-detector diffractometer	4976 independent reflections
Radiation source: fine-focus sealed tube	3284 reflections with *I* > 2σ(*I*)
graphite	*R*_int_ = 0.073
phi and ω scans	θ_max_ = 26.1°, θ_min_ = 1.8°
Absorption correction: multi-scan (*SADABS*; Sheldrick, 1996)	*h* = −17→18
*T*_min_ = 0.688, *T*_max_ = 0.721	*k* = −21→10
14065 measured reflections	*l* = −22→23

### Refinement

**Table:**

Refinement on *F*^2^	Primary atom site location: structure-invariant direct methods
Least-squares matrix: full	Secondary atom site location: difference Fourier map
*R*[*F*^2^ > 2σ(*F*^2^)] = 0.056	Hydrogen site location: inferred from neighbouring sites
*wR*(*F*^2^) = 0.113	H-atom parameters constrained
*S* = 1.02	*w* = 1/[σ^2^(*F*_o_^2^) + (0.039*P*)^2^] where *P* = (*F*_o_^2^ + 2*F*_c_^2^)/3
4976 reflections	(Δ/σ)_max_ = 0.001
262 parameters	Δρ_max_ = 0.78 e Å^−^^3^
0 restraints	Δρ_min_ = −0.42 e Å^−^^3^

### Special details

**Table:**

Geometry. All esds (except the esd in the dihedral angle between two l.s. planes) are estimated using the full covariance matrix. The cell esds are taken into account individually in the estimation of esds in distances, angles and torsion angles; correlations between esds in cell parameters are only used when they are defined by crystal symmetry. An approximate (isotropic) treatment of cell esds is used for estimating esds involving l.s. planes.
Refinement. Refinement of *F*^2^ against ALL reflections. The weighted *R*-factor wR and goodness of fit *S* are based on *F*^2^, conventional *R*-factors *R* are based on *F*, with *F* set to zero for negative *F*^2^. The threshold expression of *F*^2^ > σ(*F*^2^) is used only for calculating *R*-factors(gt) etc. and is not relevant to the choice of reflections for refinement. *R*-factors based on *F*^2^ are statistically about twice as large as those based on *F*, and *R*- factors based on ALL data will be even larger.

### Fractional atomic coordinates and isotropic or equivalent isotropic displacement parameters (Å^2^)

**Table:**

	*x*	*y*	*z*	*U*_iso_*/*U*_eq_	
Sn1	0.28085 (2)	0.63191 (2)	0.82050 (2)	0.03363 (14)	
Cl1	0.38105 (10)	0.72044 (11)	0.88005 (10)	0.0654 (6)	
C1	0.1798 (3)	0.6193 (4)	0.8932 (3)	0.0452 (17)	
H1A	0.1553	0.5691	0.8870	0.054*	
H1B	0.2087	0.6218	0.9409	0.054*	
C2	0.1012 (3)	0.6751 (3)	0.8892 (3)	0.0379 (15)	
C3	0.1392 (4)	0.7525 (4)	0.9106 (3)	0.0584 (19)	
H3A	0.0919	0.7887	0.9090	0.088*	
H3B	0.1819	0.7672	0.8782	0.088*	
H3C	0.1677	0.7500	0.9578	0.088*	
C4	0.0385 (4)	0.6530 (4)	0.9459 (3)	0.0521 (19)	
H4A	−0.0105	0.6877	0.9445	0.078*	
H4B	0.0703	0.6546	0.9919	0.078*	
H4C	0.0163	0.6031	0.9366	0.078*	
C5	0.0505 (3)	0.6738 (4)	0.8170 (3)	0.0362 (14)	
C6	0.0253 (4)	0.6067 (4)	0.7834 (3)	0.0442 (16)	
H6A	0.0417	0.5612	0.8050	0.053*	
C7	0.0247 (4)	0.7404 (4)	0.7826 (3)	0.0476 (17)	
H7A	0.0411	0.7861	0.8038	0.057*	
C8	0.3641 (3)	0.5345 (3)	0.8122 (3)	0.0376 (15)	
H8A	0.3510	0.5132	0.7653	0.045*	
H8B	0.4254	0.5516	0.8150	0.045*	
C9	0.3579 (3)	0.4711 (4)	0.8663 (3)	0.0394 (15)	
C10	0.3954 (4)	0.4993 (4)	0.9392 (3)	0.062 (2)	
H10A	0.3599	0.5405	0.9537	0.093*	
H10B	0.4556	0.5159	0.9364	0.093*	
H10C	0.3942	0.4593	0.9731	0.093*	
C11	0.4168 (4)	0.4049 (4)	0.8449 (4)	0.060 (2)	
H11A	0.4772	0.4217	0.8439	0.090*	
H11B	0.3955	0.3869	0.7988	0.090*	
H11C	0.4143	0.3649	0.8789	0.090*	
C12	0.2626 (3)	0.4417 (3)	0.8674 (3)	0.0350 (14)	
C13	0.2205 (4)	0.4316 (3)	0.9293 (3)	0.0434 (16)	
H13A	0.2508	0.4431	0.9727	0.052*	
C14	0.1342 (4)	0.4048 (3)	0.9273 (4)	0.0462 (16)	
H14A	0.1073	0.3984	0.9694	0.055*	
C15	0.0884 (4)	0.3878 (4)	0.8646 (4)	0.0567 (19)	
H15A	0.0304	0.3698	0.8639	0.068*	
C16	0.1283 (4)	0.3974 (4)	0.8018 (4)	0.0570 (19)	
H16A	0.0974	0.3861	0.7585	0.068*	
C17	0.2147 (4)	0.4239 (4)	0.8044 (3)	0.0474 (17)	
H17A	0.2414	0.4300	0.7622	0.057*	
C18	0.2457 (3)	0.6893 (3)	0.7222 (3)	0.0373 (14)	
H18A	0.1817	0.6855	0.7121	0.045*	
H18B	0.2596	0.7422	0.7291	0.045*	
C19	0.2896 (4)	0.6625 (3)	0.6566 (3)	0.0355 (14)	
C20	0.3905 (4)	0.6750 (4)	0.6689 (3)	0.0496 (17)	
H20A	0.4140	0.6446	0.7081	0.074*	
H20B	0.4022	0.7270	0.6794	0.074*	
H20C	0.4185	0.6611	0.6270	0.074*	
C21	0.2556 (4)	0.7128 (3)	0.5935 (3)	0.0483 (17)	
H21A	0.1925	0.7063	0.5843	0.072*	
H21B	0.2848	0.6988	0.5522	0.072*	
H21C	0.2683	0.7645	0.6049	0.072*	
C22	0.2664 (3)	0.5811 (3)	0.6383 (3)	0.0332 (14)	
C23	0.3288 (4)	0.5275 (4)	0.6238 (3)	0.0404 (15)	
H23A	0.3885	0.5412	0.6259	0.048*	
C24	0.3056 (5)	0.4547 (4)	0.6065 (3)	0.0501 (17)	
H24A	0.3496	0.4204	0.5969	0.060*	
C25	0.2180 (5)	0.4319 (4)	0.6032 (3)	0.0541 (18)	
H25A	0.2023	0.3825	0.5918	0.065*	
C26	0.1550 (4)	0.4837 (4)	0.6171 (3)	0.0500 (17)	
H26A	0.0956	0.4693	0.6153	0.060*	
C27	0.1778 (4)	0.5575 (4)	0.6339 (3)	0.0412 (15)	
H27A	0.1334	0.5919	0.6424	0.049*	

### Atomic displacement parameters (Å^2^)

**Table:**

	*U*^11^	*U*^22^	*U*^33^	*U*^12^	*U*^13^	*U*^23^
Sn1	0.0262 (2)	0.0405 (2)	0.0342 (2)	0.0021 (2)	0.00262 (15)	−0.0040 (2)
Cl1	0.0347 (8)	0.0812 (14)	0.0794 (13)	−0.0075 (9)	−0.0002 (8)	−0.0371 (11)
C1	0.030 (3)	0.072 (5)	0.033 (3)	0.003 (3)	0.004 (2)	0.008 (3)
C2	0.027 (3)	0.037 (4)	0.050 (4)	0.001 (3)	0.003 (3)	−0.007 (3)
C3	0.042 (4)	0.074 (5)	0.059 (4)	−0.004 (4)	0.002 (3)	−0.017 (4)
C4	0.033 (3)	0.083 (6)	0.041 (4)	−0.003 (3)	0.007 (3)	−0.006 (4)
C5	0.023 (3)	0.047 (4)	0.040 (3)	0.007 (3)	0.009 (2)	0.002 (3)
C6	0.039 (4)	0.049 (4)	0.047 (4)	0.001 (3)	0.014 (3)	0.005 (3)
C7	0.039 (4)	0.048 (4)	0.056 (4)	0.006 (3)	0.003 (3)	−0.001 (3)
C8	0.032 (3)	0.048 (4)	0.035 (3)	0.003 (3)	0.010 (3)	−0.002 (3)
C9	0.033 (3)	0.054 (4)	0.032 (3)	0.009 (3)	0.007 (3)	0.011 (3)
C10	0.047 (4)	0.094 (6)	0.044 (4)	−0.005 (4)	−0.009 (3)	0.009 (4)
C11	0.044 (4)	0.057 (5)	0.079 (5)	0.026 (4)	0.009 (4)	0.020 (4)
C12	0.031 (3)	0.033 (4)	0.041 (4)	0.007 (3)	0.003 (3)	0.002 (3)
C13	0.050 (4)	0.039 (4)	0.042 (4)	0.010 (3)	0.007 (3)	0.002 (3)
C14	0.047 (4)	0.039 (4)	0.055 (4)	0.003 (3)	0.018 (3)	0.005 (3)
C15	0.037 (4)	0.039 (4)	0.093 (6)	−0.002 (3)	0.005 (4)	−0.002 (4)
C16	0.054 (4)	0.062 (5)	0.053 (4)	0.009 (4)	−0.010 (3)	−0.008 (4)
C17	0.045 (4)	0.052 (4)	0.044 (4)	0.011 (3)	0.003 (3)	0.003 (3)
C18	0.033 (3)	0.033 (3)	0.046 (4)	−0.003 (3)	0.003 (3)	−0.002 (3)
C19	0.035 (3)	0.037 (4)	0.036 (3)	0.002 (3)	0.006 (3)	0.008 (3)
C20	0.042 (4)	0.047 (4)	0.061 (4)	−0.011 (3)	0.013 (3)	0.004 (4)
C21	0.052 (4)	0.044 (4)	0.049 (4)	−0.001 (3)	0.005 (3)	0.009 (3)
C22	0.032 (3)	0.043 (4)	0.025 (3)	0.003 (3)	0.003 (2)	0.002 (3)
C23	0.038 (3)	0.049 (4)	0.034 (3)	0.004 (3)	0.002 (3)	−0.001 (3)
C24	0.064 (4)	0.047 (4)	0.039 (4)	0.010 (4)	0.003 (3)	−0.009 (3)
C25	0.089 (5)	0.032 (4)	0.040 (4)	−0.005 (4)	−0.001 (4)	−0.002 (3)
C26	0.045 (4)	0.048 (4)	0.057 (4)	−0.020 (4)	0.002 (3)	0.004 (4)
C27	0.032 (3)	0.043 (4)	0.049 (4)	0.003 (3)	0.007 (3)	0.006 (3)

### Geometric parameters (Å, °)

**Table:**

Sn1—C1	2.146 (5)	C12—C17	1.378 (7)
Sn1—C18	2.149 (5)	C12—C13	1.387 (7)
Sn1—C8	2.152 (5)	C13—C14	1.384 (8)
Sn1—Cl1	2.3978 (17)	C13—H13A	0.9300
C1—C2	1.541 (7)	C14—C15	1.356 (8)
C1—H1A	0.9700	C14—H14A	0.9300
C1—H1B	0.9700	C15—C16	1.386 (9)
C2—C5	1.511 (7)	C15—H15A	0.9300
C2—C3	1.532 (8)	C16—C17	1.382 (8)
C2—C4	1.537 (7)	C16—H16A	0.9300
C3—H3A	0.9600	C17—H17A	0.9300
C3—H3B	0.9600	C18—C19	1.529 (7)
C3—H3C	0.9600	C18—H18A	0.9700
C4—H4A	0.9600	C18—H18B	0.9700
C4—H4B	0.9600	C19—C22	1.522 (8)
C4—H4C	0.9600	C19—C20	1.536 (7)
C5—C6	1.389 (8)	C19—C21	1.545 (7)
C5—C7	1.391 (8)	C20—H20A	0.9600
C6—C6^i^	1.422 (11)	C20—H20B	0.9600
C6—H6A	0.9300	C20—H20C	0.9600
C7—C7^i^	1.387 (11)	C21—H21A	0.9600
C7—H7A	0.9300	C21—H21B	0.9600
C8—C9	1.530 (7)	C21—H21C	0.9600
C8—H8A	0.9700	C22—C23	1.382 (7)
C8—H8B	0.9700	C22—C27	1.395 (7)
C9—C12	1.531 (7)	C23—C24	1.373 (8)
C9—C10	1.531 (8)	C23—H23A	0.9300
C9—C11	1.548 (8)	C24—C25	1.378 (8)
C10—H10A	0.9600	C24—H24A	0.9300
C10—H10B	0.9600	C25—C26	1.365 (8)
C10—H10C	0.9600	C25—H25A	0.9300
C11—H11A	0.9600	C26—C27	1.386 (8)
C11—H11B	0.9600	C26—H26A	0.9300
C11—H11C	0.9600	C27—H27A	0.9300
			
C1—Sn1—C18	117.8 (2)	H11B—C11—H11C	109.5
C1—Sn1—C8	114.3 (2)	C17—C12—C13	117.1 (5)
C18—Sn1—C8	115.0 (2)	C17—C12—C9	119.5 (5)
C1—Sn1—Cl1	102.73 (17)	C13—C12—C9	123.4 (5)
C18—Sn1—Cl1	101.32 (16)	C14—C13—C12	121.0 (6)
C8—Sn1—Cl1	102.30 (16)	C14—C13—H13A	119.5
C2—C1—Sn1	119.0 (4)	C12—C13—H13A	119.5
C2—C1—H1A	107.6	C15—C14—C13	120.8 (6)
Sn1—C1—H1A	107.6	C15—C14—H14A	119.6
C2—C1—H1B	107.6	C13—C14—H14A	119.6
Sn1—C1—H1B	107.6	C14—C15—C16	119.7 (6)
H1A—C1—H1B	107.0	C14—C15—H15A	120.1
C5—C2—C3	113.7 (5)	C16—C15—H15A	120.1
C5—C2—C4	109.4 (4)	C17—C16—C15	119.0 (6)
C3—C2—C4	106.5 (5)	C17—C16—H16A	120.5
C5—C2—C1	111.4 (5)	C15—C16—H16A	120.5
C3—C2—C1	107.2 (5)	C12—C17—C16	122.3 (6)
C4—C2—C1	108.4 (5)	C12—C17—H17A	118.8
C2—C3—H3A	109.5	C16—C17—H17A	118.8
C2—C3—H3B	109.5	C19—C18—Sn1	117.4 (4)
H3A—C3—H3B	109.5	C19—C18—H18A	107.9
C2—C3—H3C	109.5	Sn1—C18—H18A	107.9
H3A—C3—H3C	109.5	C19—C18—H18B	107.9
H3B—C3—H3C	109.5	Sn1—C18—H18B	107.9
C2—C4—H4A	109.5	H18A—C18—H18B	107.2
C2—C4—H4B	109.5	C22—C19—C18	112.0 (4)
H4A—C4—H4B	109.5	C22—C19—C20	112.2 (5)
C2—C4—H4C	109.5	C18—C19—C20	108.8 (5)
H4A—C4—H4C	109.5	C22—C19—C21	108.5 (5)
H4B—C4—H4C	109.5	C18—C19—C21	107.9 (5)
C6—C5—C7	117.5 (5)	C20—C19—C21	107.2 (5)
C6—C5—C2	121.7 (5)	C19—C20—H20A	109.5
C7—C5—C2	120.8 (6)	C19—C20—H20B	109.5
C5—C6—C6^i^	120.9 (3)	H20A—C20—H20B	109.5
C5—C6—H6A	119.6	C19—C20—H20C	109.5
C6^i^—C6—H6A	119.6	H20A—C20—H20C	109.5
C7^i^—C7—C5	121.7 (4)	H20B—C20—H20C	109.5
C7^i^—C7—H7A	119.2	C19—C21—H21A	109.5
C5—C7—H7A	119.2	C19—C21—H21B	109.5
C9—C8—Sn1	118.4 (3)	H21A—C21—H21B	109.5
C9—C8—H8A	107.7	C19—C21—H21C	109.5
Sn1—C8—H8A	107.7	H21A—C21—H21C	109.5
C9—C8—H8B	107.7	H21B—C21—H21C	109.5
Sn1—C8—H8B	107.7	C23—C22—C27	116.4 (6)
H8A—C8—H8B	107.1	C23—C22—C19	123.5 (5)
C8—C9—C12	111.7 (5)	C27—C22—C19	120.0 (5)
C8—C9—C10	108.8 (5)	C24—C23—C22	122.2 (6)
C12—C9—C10	112.2 (5)	C24—C23—H23A	118.9
C8—C9—C11	108.5 (4)	C22—C23—H23A	118.9
C12—C9—C11	107.6 (5)	C23—C24—C25	120.9 (6)
C10—C9—C11	107.8 (5)	C23—C24—H24A	119.6
C9—C10—H10A	109.5	C25—C24—H24A	119.6
C9—C10—H10B	109.5	C26—C25—C24	118.1 (6)
H10A—C10—H10B	109.5	C26—C25—H25A	120.9
C9—C10—H10C	109.5	C24—C25—H25A	120.9
H10A—C10—H10C	109.5	C25—C26—C27	121.3 (6)
H10B—C10—H10C	109.5	C25—C26—H26A	119.4
C9—C11—H11A	109.5	C27—C26—H26A	119.4
C9—C11—H11B	109.5	C26—C27—C22	121.1 (6)
H11A—C11—H11B	109.5	C26—C27—H27A	119.5
C9—C11—H11C	109.5	C22—C27—H27A	119.5
H11A—C11—H11C	109.5		
			
C18—Sn1—C1—C2	−23.9 (5)	C17—C12—C13—C14	0.0 (9)
C8—Sn1—C1—C2	−163.6 (4)	C9—C12—C13—C14	−180.0 (5)
Cl1—Sn1—C1—C2	86.4 (4)	C12—C13—C14—C15	−0.1 (9)
Sn1—C1—C2—C5	58.8 (6)	C13—C14—C15—C16	−0.1 (10)
Sn1—C1—C2—C3	−66.2 (6)	C14—C15—C16—C17	0.3 (10)
Sn1—C1—C2—C4	179.2 (4)	C13—C12—C17—C16	0.2 (9)
C3—C2—C5—C6	168.2 (5)	C9—C12—C17—C16	−179.8 (6)
C4—C2—C5—C6	−72.9 (7)	C15—C16—C17—C12	−0.4 (10)
C1—C2—C5—C6	46.9 (7)	C1—Sn1—C18—C19	−145.8 (4)
C3—C2—C5—C7	−13.9 (7)	C8—Sn1—C18—C19	−6.4 (5)
C4—C2—C5—C7	105.0 (6)	Cl1—Sn1—C18—C19	103.1 (4)
C1—C2—C5—C7	−135.2 (5)	Sn1—C18—C19—C22	61.7 (5)
C7—C5—C6—C6^i^	−0.3 (10)	Sn1—C18—C19—C20	−62.9 (6)
C2—C5—C6—C6^i^	177.7 (6)	Sn1—C18—C19—C21	−179.0 (4)
C6—C5—C7—C7^i^	0.4 (10)	C18—C19—C22—C23	−131.9 (5)
C2—C5—C7—C7^i^	−177.6 (6)	C20—C19—C22—C23	−9.2 (7)
C1—Sn1—C8—C9	−11.0 (5)	C21—C19—C22—C23	109.1 (6)
C18—Sn1—C8—C9	−151.8 (4)	C18—C19—C22—C27	49.5 (7)
Cl1—Sn1—C8—C9	99.3 (4)	C20—C19—C22—C27	172.2 (5)
Sn1—C8—C9—C12	56.1 (6)	C21—C19—C22—C27	−69.5 (6)
Sn1—C8—C9—C10	−68.3 (5)	C27—C22—C23—C24	−0.5 (8)
Sn1—C8—C9—C11	174.6 (4)	C19—C22—C23—C24	−179.1 (5)
C8—C9—C12—C17	48.6 (7)	C22—C23—C24—C25	−0.3 (9)
C10—C9—C12—C17	171.1 (6)	C23—C24—C25—C26	0.4 (9)
C11—C9—C12—C17	−70.4 (7)	C24—C25—C26—C27	0.3 (9)
C8—C9—C12—C13	−131.4 (6)	C25—C26—C27—C22	−1.1 (9)
C10—C9—C12—C13	−8.9 (8)	C23—C22—C27—C26	1.1 (8)
C11—C9—C12—C13	109.6 (6)	C19—C22—C27—C26	179.8 (5)

Symmetry codes: (i) −*x*, *y*, −*z*+3/2.
